# Polypharmacy and potentially-inappropriate medications are prevalent in the elderly cancer patients receiving systemic cancer therapy and they co-relate with adverse outcomes

**DOI:** 10.1186/s12877-023-04471-3

**Published:** 2023-11-27

**Authors:** Chanyoot Bandidwattanawong, Pat Rattanaserikulchai, Nontakorn Jetsadavanit

**Affiliations:** 1 Division of Medical Oncology, Department of Internal Medicine, Faculty of Medicine, Navamindradhiraj University, Bangkok, Thailand; 2 Department of Internal Medicine, Faculty of Medicine, Navamindradhiraj University, Bangkok, Thailand

**Keywords:** Elderly cancer patients, Polypharmacy, Potentially inappropriate medications, Prevalence, Adverse outcomes

## Abstract

**Objectives:**

Cancer is the disease of the ageing. Most of the elderly cancer patients have pre-existing illnesses requiring complexity of medical care. Excessive medications would lead not only futility, but also result in adverse outcomes especially if such over-prescription is not appropriate. This study was intended to determine the prevalence of polypharmacy (PP) and potentially-inappropriate medications (PIMs) among elderly cancer patients eligible for active cancer care and their associations with hospitalization and mortality.

**Materials and methods:**

This was a prospective cohort study conducted among the elderly non-hematologic cancer patients (≥ 65 years old) whom a medical oncologist had decided suitable for systemic cancer therapy. Demographic data including age, sex, primary site of cancer, cancer stage at diagnosis, Charlson Comorbidity Index (CCI), numbers and kinds of medications used both prior to and during cancer treatment were recorded. Hospitalizations not related to systemic cancer therapy administration and mortality were prospectively monitored. All of the patients had to be followed at least one year after cancer diagnosis.

**Results:**

There were 180 eligible participants. Median age in years (IQR) was 68 (65–73). One hundred patients (55.56%) were male and 80 patients (44.44%) were female. Breast (35, 19.44%), lung (31, 17.22%) and colorectal (18, 10%) cancers were the most common diagnoses. Eighty-six patients (47.78%) had metastatic disease at cancer diagnosis. One hundred twenty-two patients (67.78%) had PP (5 or more medications a day) and thirty-six patients (20%) had hyper-PP (10 or more medications a day). One hundred twenty five of the whole cohort (69.4%) had PIMs. Patients with more serious CCI scores were associated with PP and hyper-PP. While patients with primary lung cancer was only the only factor associated with PIMs. When excluding opioids, laxatives and anti-emetics, the most frequently prescribed drugs during cancer treatment, the so-called corrected PP did not associate with worse 1-year survival. Factors correlated with 1-year mortality were more advanced age group (70 years old or more) (OR 2.24; 95% C.I., 1.14–4.41; *p* = 0.019), primary lung cancer (OR 2.89; 95% C.I., 1.45–5.78; *p* = 0.003), metastatic disease at cancer diagnosis (OR 4.57; 95% C.I., 1.90–10.97; *p* = 0.001), and unplanned hospitalizations (OR 3.09; 95% C.I.,1.60–5.99; *p* = 0.001). While male gender (OR 2.35; 95% C.I., 1.17–4.71; *p* = 0.016), metastatic stage at cancer diagnosis (OR 2.74; 95% C.I., 1.33–5.66; *p* = 0.006) and corrected PP (OR 1.90; 95% C.I. 1.01–3.56; *p* = 0.046) were the significant predictive factors of unplanned hospitalizations.

**Conclusion:**

Among elderly cancer patients suitable for systemic cancer therapy, around two thirds of patients had PP and PIMs. Higher CCI score was the only significant predictor of PP and hyper-PP; while primary lung cancer was the sole independent factor predicting PIMs. PP was associated with unplanned hospitalizations, albeit not the survival.

**Supplementary Information:**

The online version contains supplementary material available at 10.1186/s12877-023-04471-3.

## Introduction

Polypharmacy (PP), as defined by Hajjar et al*.* that “the use of multiple medications and/or the administration of more medications than are clinically indicated and representing unnecessary drug use” [1] is one of the most concerning health care issue. It is a ubiquitous problem in any age group; however, it is especially a serious trouble for the elderly patients who tend to consume more drugs than younger patients do. It has been reported that the more patient ages, the larger number of medications are prescribed [2]. Understandably, it is as a result of increasing co-morbid conditions prevalent in this population. Jörgensen, et al*. *reported that 78% of patients older than 65 years were consistently on drugs, and that 39% regularly took five or more drugs. The most commonly used pharmacologic groups included cardiovascular, nervous system, and gastrointestinal medications [3]. Among Asian population, the recent study by Cho, et al*. *demonstrated that around 47.8% of the elderly had PP (as defined as more than 5 medications a day) and 11.9% had hyper-PP (more than 10 medications a day) for more than 90 days. Male sex, older age, insurance, co-morbidities (cardio-cerebrovascular disease, diabetes mellitus, depressive disorder, dementia, an Elikhauser comorbidity index of more than 3), and healthcare service utilization were associated with an increased probability of polypharmacy. Drugs for acid-related disorders were most commonly prescribed [4]. Depending on the definition of polypharmacy, 11% to 96% of the elderly cancer patients were subjected to be exposed too many concurrent medications [5]. Jörgensen, et al*. *conducted a population-based case–control study to explore pattern of drug uses among Danish cancer patients and found that at cancer diagnosis, 35% of elderly cancer patients used more than 5 drugs daily compared with 27% of controls. Analgesics, acid-suppressing drugs, and antibiotics were among the most commonly prescribed medications up to six months prior to their cancer diagnoses [6]. Nightingale, et al*. *retrospectively examined medication use among the elderly cancer patients in ambulatory setting and reported the prevalence of PP and hyper-PP of 84% and 43%, respectively [7]. Corresponding to the prevalence in Western countries, evidences from Asian countries also showed substantially higher prevalence of PP among the elderly cancer population. Yeoh, et al*. *reported 58% of the elderly cancer patients in Singapore had polypharmacy. Moreover, compared to those elderly cancer patients without PP, those with PP had higher significant co-morbidities assessed using the Charlson co-morbidity index [8]. Takemoto, et al*. *showed that 23.1% and 32.6% of the elderly Japanese cancer patients in curative and palliative settings, respectively had polypharmacy and the increased number of medications was associated with the progression of cancer [9].

Besides medical futility, PP, especially with potentially in‐appropriate medications (PIMs) was associated with risk of toxicities from cancer treatment, treatment discontinuation [10–12], unplanned hospitalizations [13] and death among elderly cancer patients [14]. Since PP is associated with frailty syndrome, poor physical function, and a significantly higher major co-morbidities [7, 15], it is difficult to determine PP as a contributing factor of poor survival among elderly cancer patients. This study was intended to assess the prevalence of PP and PIMs and evaluate which factors were associated with both conditions; moreover, the investigators intended to determine whether PP and PIMs were associated with unplanned hospitalizations and mortality.

## Patients and methods

This study was an observational prospective cohort study conducted in Division of Medical Oncology, Department of Internal Medicine, Faculty of Medicine Vajira Hospital, Navamindradhiraj University, Bangkok, Thailand. Eligible patients were cancer patients aged more than 65 years old who had recently diagnosed to have solid malignancies (excluding hematologic cancers). Those whom a medical oncologist had decided to treat with systemic cancer treatments (cytotoxic agents or targeted therapies or both) were enrolled. Those who deserved only supportive or end of life care only due to any reasons (flail or unfitted conditions as a result of serious pre-existing condition(s), or patient and/or his caregivers’ intents of no aggressive cancer management) were excluded. Those who had not attended regular visits during the first year after cancer diagnosis were also excluded. The investigators provided the information to all of the participants regarding the necessities, benefits and any inconveniences during conducting this research prior to asking them for the informed consent. After signing the informed consent, demographic data regarding age, sex, primary site of cancer, cancer stage at diagnosis, and pre-existing medical conditions were collected from a participant’s electronic medical records. The medication review included *medications prior to cancer diagnosis* defined as any medications prescribed from two weeks to one year before cancer diagnosis and *medications after cancer diagnosis* defined as any medications prescribed concomitant with active cancer treatment. The maximal numbers of such medications at any time since one year prior to cancer diagnosis and during active cancer care were recorded and used as whether participants had polypharmacy and potentially inappropriate medications (PIMs). According to a systemic review by Masnoon, et al. [16], the most commonly reported definition of polypharmacy was the numerical definition of five or more medications daily, therefore the investigators defined *polypharmacy (PP)* as more than 5 medications daily excluding anti-cancer drugs (cytotoxic agents and/or targeted therapies) and defined *hyper-polypharmacy (hyper-PP)* as more than 10 medications daily excluding anti-cancer drugs. Since most of the enrolled patients requiring at least one of the following kinds of medications, opioid analgesics, laxatives and anti-emetic agents, the sensitivity analysis was also performed to determine *corrected PP* by exclusion of opioid analgesics, laxatives and anti-emetic agents. Potentially inappropriate medications (PIMs) was categorized using the 2019 Beers criteria [17], endorsed by the American Geriatrics Society. Every eligible participant was progressively followed up at least 1 year to determine further concomitant medications, *unplanned hospitalization* (defined as any hospitalizations due to any causes except from anti-cancer treatment administration or in-patient clinical investigations such as imaging studies and endoscopic examinations scheduled to be obtained un-related to a patient’s worsening clinical condition) and survival. The degree of seriousness of pre-existing medical conditions was evaluated by Charlson co-morbidity index (CCI). The survival outcome was determined using *1-year survival rate* defined as the proportion of patients who survived 1-year after cancer diagnosis. The survival time was started from the date of first oncological clinic visit. The sample size was calculated based on the study by Khaledi AR et al. [18], with expected drop-out rate of 10%, the final expected number of participants was at least 163. Enrollment of participants occurred between January 1, 2021 to December 1, 2022. The study was conducted according to the guidelines of the Declaration of Helsinki, and was approved by the Institutional Review Board of Faculty of Medicine Vajira Hospital (COA 022/2565). All of the participants were informed both verbally and literally prior to sign consent forms.

The primary outcome was the prevalence of PP in cancer patients suitable for systemic cancer therapy. The secondary outcomes were the prevalence of hyper-PP, associations between PP and other baseline demographics, rate of unexpected hospitalization and 1-year survival. Also the secondary outcomes were the prevalence of PIMs. Descriptive analyses were performed to evaluate variables related to PP, hyper PP, PIMs, unexpected hospitalizations and 1-year survival. Median and inter-quartile range (IQR) were generated for continuous data, and proportions and frequencies for categorical data. Bivariate associations between baseline variables, PP/PIMs, unexpected hospitalization and 1-year survival rate were calculated by using Fisher’s exact test and Pearson’s χ^2^ test for categorical variables and independent *t*-test for continuous variable. Logistic regression was used to calculate unadjusted and adjusted odds ratios (ORs) and 95% confidence intervals (C.I.s) for the association between PP and various clinical parameters. A dichotomous PP variable (≥ 5 vs < 5 drugs and ≥ 10 vs < 10)) was used in analyses for associations of baseline demographic data with PP. The associations of PP (analyzed as a variable with three categories: 0–4 drugs as reference group; 5–9 drugs; and ≥ 10 drugs) and unexpected hospitalizations and 1-year survival were assessed with Cox proportional hazards models. The investigators also carried out the sensitivity analysis using *corrected PP* as defined as PP in exclusion of opioid analgesics, laxatives and anti-emetics. The proportional hazards assumption was assessed for the main determinants and all covariates by adding time-dependent interaction terms. Two-sided *p*-values of < 0.05 were considered statistically significant. Statistical analysis was performed by using STATA/IC Software, Version 17.0 (Stata Corp, College Station, TX, USA).

## Results

There were 236 elderly patients with non-hematologic cancer aged more than 65 years old, 198 of them were eligible for systemic cancer treatment and 18 of them were later excluded due to loss to follow up during a year after cancer diagnosis. Table 1 showed baseline characteristics of the participants. There were 180 eligible participants. Median age (IQR) was 68 (65–73) years old. One hundred patients (55.56%) were male and 80 patients (44.44%) were female. Breast (19.44%), lung (17.22%) and colorectal (10%) cancers were the most common diagnoses. Nearly half of them (86 of the 180 patients, 47.78%) had metastatic stage at cancer diagnosis. One hundred twenty-two (122) of the total 180 patients (67.78%) of the elderly cancer patients eligible for active oncological treatment had PP. Notably, thirty-six of the whole cohort (20%) had hyper-PP (more than 10 medications per day). The median number of prescribed pre-cancer diagnosis medications in PP group was 4.5 (IQR, 2–8) and escalated to median of 7 (IQR, 6–10) after cancer diagnosis. Compared to non-PP group, the PP group had worse Charlson Co-morbidity Index (CCI) (median of 8 (IQR, 5–9)), the majority (59.8%) of patients were male and primary lung cancer was the most common diagnosis (19.7%). Besides acetaminophen, opioid analgesics, laxatives, and anti-emetics, the most-commonly prescribed medications during active cancer treatment were cardiovascular agents (including anti-hypertensive, anti-platelets, anti-coagulants), proton-pump inhibitors and antibiotics (Supplement 1).

**Table 1:** Baseline characteristics of participants

	**Total**	**non-Polypharmacy (< 5)**	**Polypharmacy (> 5)**
	***n* = 180**	***n* = 58**	***n* = 122**
**Age (years) (Median (IQR))**	68 (65–73)	68 (65–71)	68.5 (65–74)
**Male:Female (N (%))**	100 (55.56): 80 (44.44)	27 (46.6): 31 (53.4)	73 (59.8): 49 (40.2)
**Type of Cancer (N (%))**			
CA lung	31	7 (12.1%)	24 (19.7%)
CA breast	35	19 (32.8%)	16 (13.1%)
CA colon	18	7 (12.1%)	11 (9%)
CA rectum	10	5 (8.6%)	5 (4.1%)
HCC	11	3 (5.2%)	8 (6.6%)
CA stomach	2	1 (1.7%)	1 (0.8%)
CA pancrease	6	2 (3.4%)	4 (3.3%)
CA bladder	16	2 (3.4%)	14 (11.5%)
CA prostate	7	0 (0%)	7 (5.7%)
CA esophagus	8	2 (3.4%)	6 (4.9%)
CA head and neck	17	4 (6.9%)	13 (10.7%)
Sarcoma	4	4 (6.9%)	0 (0%)
Melanoma	1	1 (1.7%)	0 (0%)
Cholangiocarcinoma	4	1 (1.7%)	3 (2.5%)
Other	10	0 (0%)	10 (8.2%)
**Stage (N (%))**			
Stage 1–3	94	34 (58.6%)	60 (49.2%)
Stage 4	86	24 (41.4%)	62 (50.8%)
**Charlson Co-morbidity Index (Median (IQR))**	8 (5–9)	5 (4–8)	8 (5–9)
**Number of Medications (Median (IQR))**			
Prior to cancer diagnosis	3 (0–6.5)	1 (0–2)	4.5 (2–8)
After cancer diagnosis	6 (4–8)	3 (2–4)	7 (6–10)

We also determined the prevalence of PIMs and revealed that around 69.4% (125 of the 180 patients) of the participants exposed to inappropriate prescriptions. The most commonly inappropriate prescribed medications were PPIs, benzodiazepines (BZPs), peripheral alpha-1 inhibitors (e.g. doxazosin, alfuzosin), muscle relaxants and various psycho-active agents (Supplement 2).

To explore the factors associated with PP, we found that more serious co-morbidity, in terms of higher Charlson Co-morbidity Index (CCI) (5 or more) was the sole independent predictive factor. The investigators also performed the sensitivity analysis by excluding the top three classes of the most commonly prescribed medications during active cancer treatment i.e. opioids, laxatives and anti-emetics, the CCI remained the sole significant predictor of PP (Tables 2 and 3). When we used the hyper-PP in the model, the CCI still remained the sole significant predictor (The data was not shown.). On the contrary, primary lung cancer was the only independent predictor of PIMs (Table 4).

**Table 2:** Association between polypharmacy and baseline characteristics

	**Non-Polypharmacy(< 5)**	**Polypharmacy (≥ 5)**	**Adjusted**	**95%CI**	***P*-value**
	***n* = 58**	***n* = 122**	**Odds ratio**^a^		
**Age**					
< 70	43 (35.8%)	77 (64.2%)	1		
> = 70	15 (25%)	45 (75%)	1.13	0.52–2.48	0.759
**Gender**					
Male	27 (27%)	73 (73%)	1.56	0.81–3.01	0.188
Female	31 (38.8%)	49 (61.3%)	1		
**Type of cancer**					
Non-pulmonary cancer	51 (34.2%)	98 (65.8%)	1		
Primary pulmonary cancer	7 (22.6%)	24 (77.4%)	1.75	0.69–4.46	0.240
**Stage**					
Stage 1–3	34 (36.2%)	60 (63.8%)	1		
Stage 4	24 (27.9%)	62 (72.1%)	0.91	0.42–1.95	0.801
**CCI**					
< 5	20 (54.1%)	17 (45.9%)	1		
> = 5	38 (26.6%)	105 (73.4%)	3.09	1.19–8.01	0.021

^a^Logistic regression

**Table 3:** Association between corrected polypharmacy and baseline characteristics

	**non-Polypharmacy (< 5)**^a^	**Polypharmacy (> 5)**^a^	**Adjusted**	**95%CI**	***P*-value**
	***n* = 106**	***n* = 74**	**Odds ratio**^b^		
**Age**					
< 70	76 (63.3%)	44 (36.7%)	1		
> = 70	30 (50%)	30 (50%)	1.20	0.60–2.40	0.606
**Gender**					
Male	56 (56%)	44 (44%)	1.16	0.62–2.18	0.644
Female	50 (62.5%)	30 (37.5%)	1		
**Type of cancer**					
Non-pulmonary cancer	90 (60.4%)	59 (39.6%)	1		
Primary pulmonary cancer	16 (51.6%)	15 (48.4%)	1.50	0.66–3.38	0.331
**Stage**					
Stage 1–3	56 (59.6%)	38 (40.4%)	1		
Stage 4	50 (58.1%)	36 (41.9%)	0.64	0.32–1.26	0.192
**CCI**					
< 5	30 (81.1%)	7 (18.9%)	1		
> = 5	76 (53.1%)	67 (46.9%)	4.41	1.57–12.34	0.005

^a^Excluding opioid analgesics, laxatives, and anti-emetics

^b^Logistic regression

**Table 4:** Association between PIMs and baseline characteristics

	**No PIM**	**PIMs**	**Adjusted**	**95%CI**	***P*-value**
	***n* = 55**	***n* = 125**	**Odds ratio**^a^		
**Age**					
< 70	39 (32.5%)	81 (67.5%)	1		
> = 70	16 (26.7%)	44( 73.3%)	0.87	0.40–1.92	0.735
**Gender**					
Male	29 (30.9%)	65 (69.1%)	1.06	0.55–2.07	0.857
Female	26 (30.2%)	60( 69.8%)	1		
**Type of cancer**					
Non-primary lung cancer	18 (48.6%)	19 (51.4%)	1		
Primary pulmonary cancer	37 (25.9%)	106 (74.1%)	1.29	1.52–3.19	0.006
**Stage**					
Stage 1–3	47 (31.5%)	102 (68.5%)	1		
Stage 4	8 (25.8%)	23 (74.2%)	0.57	0.26–1.26	0.163
**CCI**					
< 5	29 (29%)	71 (71%)	1		
> = 5	26 (32.5%)	54 (67.5%)	3.96	0.48–10.65	0.583

^a^Logistic regression

We further conducted the exploratory analyses to determine factors related to 1-year mortality and unplanned hospitalizations. Table 5 showed the associated factors of 1-year mortality. We used corrected PP (excluding opioid analgesics, laxatives and anti-emetics) in the model and found that factors correlated with 1-year mortality were more advanced age group (70 years old or more) (OR 2.24; 95% C.I.,1.14–4.41; *p* = 0.019), primary lung cancer (OR 2.89; 95% C.I.,1.45–5.78; *p* = 0.003), metastatic disease at cancer diagnosis (OR 4.57; 95% C.I.,1.90–10.97; *p* = 0.001), and unplanned hospitalizations (OR 3.09; 95% C.I.,1.60–5.99; *p* = 0.001). The corrected PP did not associate with 1-year survival. We also analyzed in another model including hyper-PP (0–4, 4–9, ≥ 10) as a variable factor; however, the result remained unchanged. Both corrected PP and hyper-PP did not associate with the worse 1-year mortality rate (the data was not shown). As shown in Table 6, we determined the correlation with the unplanned hospitalizations and demonstrated that male gender (OR 2.35; 95% C.I., 1.17–4.71; *p* = 0.016), metastatic stage at cancer diagnosis (OR 2.74; 95% C.I., 1.33–5.66; *p* = 0.006) and corrected PP (OR 1.90; 95% C.I. 1.01–3.56; *p* = 0.046) were the significant predictive factors. The causes of hospitalization were mainly due to presumed serious infections including sepsis, both related and un-related to cancer treatment-induced neutropenia.

**Table 5:** Factors associated with 1-year mortality

	**Survived**		**Died**		**Un-adjusted**	**95%CI**	***P*-value**	**Adjusted**	**95%CI**	***P*-value**
	**n**	**%**	**n**	**%**	**OR**			**OR**^a^		
**Age**										
< 70	98	81.7	22	18.3	1			1		
> = 70	41	68.3	19	31.7	1.82	0.99–3.37	0.06	2.24	1.14–4.41	0.019
**Gender**										
Male	73	73	27	27	1.61	0.85–3.08	0.15	1.25	0.65–2.44	0.504
Female	66	82.5	14	17.5	1			1		
**Type of cancer**										
Non-primary lung cancer	121	81.2	28	18.8	1			1		
Primary lung cancer	18	58.1	13	41.9	2.58	1.34–4.99	0.005*	2.89	1.45–5.78	0.003
**Stage**										
Stage 1–3	86	91.5	8	8.5	1			1		
Stage 4	53	61.6	33	38.4	5.45	2.51–11.80	< 0.001*	4.57	1.90–10.97	0.001
**CCI**										
< 5	35	94.6	2	5.4	1			1		
> = 5	104	72.7	39	27.3	5.70	1.38–23.60	0.016*	1.19	0.23–6.17	0.840
Polypharmacy*										
< 5	85	80.2	21	19.8	1			1		
> = 5	54	73	20	27	1.41	0.76–2.60	0.27	0.95	0.49–1.83	0.875
**Unplanned hospitalizations**										
No	117	84.8	21	15.2	1			1		
Yes	22	52.4	20	47.6	3.84	2.08–7.10	< 0.001*	3.09	1.60–5.99	0.001

^*^Excluding opioid analgesics, laxatives and anti-emetics

^a^Cox’s Proportional Hazard Model

**Table 6:** Factors associated with unplanned hospitalizations

	**No unplanned hospitalization**		**Unplanned hospit hospitalization**		**Unadjusted**	**95%C.I**	***P*-value**	**Adjusted**	**95%C.I**	***P*-value**
	**n**	**%**	**n**	**%**	**OR**			**OR**^a^		
**Age**										
< 70	94	78.3	26	21.7	1			1		
> = 70	44	73.3	16	26.7	1.39	0.74–2.59	0.30	1.27	0.64–2.53	0.488
**Gender**										
Male	69	69	31	31	2.45	1.23–4.88	0.01*	2.35	1.17–4.71	0.016
Female	69	86.3	11	13.8	1			1		
**Type of cancer**										
Non-primary lung cancer	115	77.2	34	22.8	1			1		
Primary lung cancer	23	74.2	8	25.8	1.43	0.66–3.10	0.36	1.40	0.63–3.12	0.412
**Stage**										
Stage 1–3	79	84	15	16	1			1		
Stage 4	59	68.6	27	31.4	2.65	1.41–4.98	0.003*	2.74	1.33–5.66	0.006
**CCI**										
< 5	32	86.5	5	13.5	1			1		
> = 5	106	74.1	37	25.9	2.33	0.92–5.93	0.08	0.87	0.28–2.71	0.803
Corrected polypharmacy *										
< 5	88	83.0	18	17.0	1			1		
> = 5	50	67.6	24	32.4	2.03	1.10–3.74	0.02*	1.90	1.012–3.557	0.046

^*^Excluding opioid analgesics, laxatives, and anti-emetics

^a^Cox’s Proportional Hazard Model

We deduced that more fragile patients as represented by extreme age, primary lung cancer, metastatic disease at cancer diagnosis, and unplanned hospitalizations were the most vulnerable subgroups that an oncologist should determine specific cancer management meticulously. Furthermore, besides male patients and metastatic disease at cancer diagnosis, corrected PP had strong tendency to lead to potential serious adverse events from cancer treatment as shown by unplanned hospitalizations.

## Discussion

Relying on the definition of PP, from 11 to 96% of the elderly cancer patients were exposed to excessive medications [5]. This study practically defined PP in numerical term; however, duration of continuing medications would have been more logical to be determined, if long-term association with adverse events were the outcome of interest. The prevalence of PP and hyper-PP of 67.8% and 20%, respectively were not doubtfully outstanding compared with those reported by studies conducted among Asian populations. Khaledi, et al. reported the strikingly 88% frequency among cancer patients unselected by age group [18]. Yeoh, et al*. *reported 58% of the elderly cancer patients in Singapore had PP [8]. Takemoto, et al*. *showed that 23.1% and 32.6% of the elderly Japanese cancer patients in curative and palliative settings, respectively had PP [9]. The most recent data from the Western countries was reported by Ramsdale, et al*.*She and her colleagues collected data from a randomized study enrolling patients aged ≥ 70 years with advanced cancer conducted at University of Rochester Cancer Center. Among 718 patients (mean age 77.6 years), PP (≥ 5 medications) and hyper-PP (≥ 10 medications) were identified in 61.3% and 14.5%, respectively [19]. We also found that PP and hyper-PP were both associated with existing co-morbidities; nevertheless, age (as differentiated between less than and more than 70 years old), sex, staging, type and cancer stage were not. As determined by Charlson Co-morbidity Index (CCI), the more serious co-morbidities a patient had, the higher number of concomitant medications were prescribed. The association between PP and co-morbidity was still existed when the sensitivity analysis was performed by exclusion of opioid analgesics, laxatives and anti-emetics. Turner, et al*. *evaluated the prevalence and factors associated with PP in the elderly Australian cancer patients and demonstrated that when adjusting for age, sex, instrumental activities of daily living (IADLs), Karnofsky Performance Scale (KPS), physical function (using SF-36), pain (using 10-point visual analogue scale, VAS), exhaustion (using CES-D) and distress (using 10-point VAS), polypharmacy was remained independently associated with higher CCI scores [15]. Our study excluded the elderly patients who had not received active anti-cancer management mainly due to an oncologist’s discretion. Presumably, most of them were either frail or unfitted for aggressive cancer care. Therefore, we concluded that CCI was still independently associated with PP and hyper-PP among elderly cancer patients fitted for aggressive cancer management. The study by Yeoh, et al*. *(still presented in abstract) also demonstrated the same co-relation [8]. Cardiovascular agents (including anti-hypertensive, anti-platelets, anti-coagulants and miscellaneous), proton-pump inhibitors and antibiotics were the top pharmacological classes most frequently prescribed in this cancer patients’ cohort. In accordance with a recent study in the USA, Ramsdale, et al*. *reported that cardiovascular drugs were the most prevalent in the elderly cancer patients [20]. Nightingale, et al*. *also mentioned that among ambulatory elderly cancer cares, the prevalence of PP was high and cardiovascular drugs, anti-lipidemic drugs and drugs modulating GI system were the most prevalent ones [7]. Earlier studies reporting the prevalence in Denmark showed that analgesics, acid-suppressing drugs, and antibiotics were most frequently prescribed up to six months preceding cancer diagnosis [6]. Among studies investigating the consequences of PP in the broader populations, Guthrie, et al*. *showed that cardiovascular drugs, and drugs affecting the CNS and GI systems were the most frequent prescribed in a Scottish community and they also speculated that not only the prevalence of PP was rising, but the prescribed drug combinations would be more complicated and potentially harmful [20]. A warning signal from a South Korean study conducted by Cho, et al*. *would represent the burden of PP in Asian countries. Cho and colleagues found that nearly half of the elderly South Korean patients had PP and the combinations of cardiovascular, anti-lipidemic and acid-suppressing drugs were most commonly prescribed [4]. Based on these evidences, there has been the global trend towards more and more perplexed prescriptions of the drug classes affecting or modulating cardiovascular and nervous systems representing the burdens of non-communicable diseases (NCDs) in the ageing society emerging in many parts of the world.

As a result of the difficulty in determining serious potentially clinically significant drug-drug interactions (PDIs), we evaluated the association between PP/PIMs and unplanned hospitalizations instead. We found that both corrected PP (excluding opioids, laxatives and anti-emetics) and hyper-PP, male gender and metastatic disease at cancer diagnosis were the independent factors associated with unplanned hospitalizations. Interestingly, the causes of hospitalization were fundamentally related to infections, both associated with and un-associated with cancer-treatment-induced neutropenia. However, due to non-specific presentations among the elderly patients, altered consciousness presumptively diagnosed to have sepsis would rather be the manifestations of undesired side effects from drug interactions from excessive medications used. Due to the ever-changing numbers of prescribed medications and their exact duration of truly administered drugs, we found inconclusive effect of PIMs to survival and hospitalizations. There were incoherent evidences demonstrating the detrimental effects of PP and PIMs among cancer patients. Sehgal et al*. *performed a retrospective analysis in a general patient population and found that the presence of concomitant PIMs and PP had a statistically significant tendency to increased hospital readmissions; nevertheless, PIMs alone was not significantly correlated [21]. Maggiore et al*. *conducted a secondary analysis of a prospective study to determine the prevalence and the effect of PP and PIMs on chemotherapy-related adverse events (AEs) and revealed no meaningful association found between either PP or PIMs and AEs or hospitalization. They also further explored the consequence of taking one or more of the 6 high-risk pharmacologic classes of medications potentially related to serious AEs (i.e., anti-coagulants, anti-platelet agents, opioids, insulin, oral hypoglycemics and anti-arrhythmics) and again they found no specific pharmacologic class significantly associated with either outcome [22]. Sganga, et al*. *carried out a prospective cohort study conducted in elderly patients (not cancer patients in particular) who had discharged from acute care hospitals to determine the rate of re-hospitalization and mortality within 1 year after discharge also demonstrated that multiple-drug uses (defined as more than 8 drugs a day) was at increased risk of re-hospitalization [13]. A systemic review and meta-analysis by Mohamed MR et al. concluded that even though various definitions of PP, heterogeneities in terms of both study designs and populations, PP was associated with post-operative complications, chemotherapy toxicities and both physical and functional decline [10].

Regarding the association between PP/PIMs and survival, there were scant evidences intended to determine the effect of PP/PIMs on survival in particular. We demonstrated that more advanced age (≥ 70 years), primary lung cancer, metastatic disease at cancer diagnosis, unexpected hospitalizations were the significant predictive factors of 1-year mortality. However, we failed to demonstrate both PP and hyper-PP with the mortality. Elderly patients with primary lung cancer and advanced stage at diagnosis were unquestionably vulnerable subgroups requiring properly tailoring cancer management. More extreme age and unplanned hospitalizations were the factors to be elucidated. Even though cancer is the disease of ageing; however, age alone should not be the solitary factor of exclusion of ageing patients from active cancer treatment. Co-morbidities and ageing exist independently and the prevalence of co-morbid conditions climbs with increasing age [23]. Numerous data have emphasized on assessing biological rather than chronological age in individual cancer patients. The comprehensive geriatric assessment (CGA) can provide extensive information of both functional and physiological age of an elderly person with cancer in particular. Several domains, including physical function, cognition, nutrition, comorbidities, psychological status, and social support are evaluated together. Although it consumes clinical visiting time, its results can guide a physician to choose proper management more systemically [24, 25]. The multi-comorbidity was not the independent factor for survival in this elderly cancer patients’ cohort; however, a probability of the fact that some co-morbid conditions were more predictive than the others cannot be excluded. Provocatively, a real-world data reported by Karuturi et al*. *supported the drawback of PP/PIMs on mortality as our results did. They analyzed a substantially large elderly cancer patients’ cohort including 1595 breast cancer patients and 1528 colorectal cancer patients from the SEER database. They demonstrated that among elderly breast cancer patients, 37.5% had 1 or more adverse outcomes (emergency room (ER) visit, hospitalization or death). PP, advanced stage, higher co-morbidity, and prior ER visits/hospitalizations were significantly associated with such adverse outcomes. PIMs (defined as using any drugs included in DAE list) was associated with an increased risk of death. In line with the elderly breast cancer cohort, 45% of the elderly colorectal cancer patients had at least 1 adverse outcome. Again PP, more older age, female sex, and higher co-morbidity were the independent factors. Confusingly, baseline PIMs did not co-relate with time to any events of adverse outcomes [26]. Whether PP and PIMs are associated with the mortality in the elderly cancer patients receiving aggressive oncological management is a subject to debate. We noticed that secondary data from randomized clinical trials would not represent the realistic point of view because clinical trials usually recruit highly-selective participants compared to the real world practices do. Recent GAP70 + randomized trial demonstrated that applying the geriatric assessment intervention could ameliorate serious adverse events, falls and polypharmacy in elderly patients with advanced cancer patients receiving oncological treatment including chemotherapy [27]. The investigators suggest that among such vulnerable patients, judicious and more vigilant prescriptions are advocated. Such strategy will lead to reduction in medical futility and avoidance of unexpected adverse events.

In conclusion, we revealed the prevalence of PP among elderly cancer patients eligible for systemic cancer treatment was 67.8% and the prevalence of PIMs of 69.4% was also unacceptably high. We speculated that the number of prescribed drugs depended on a pre-existing patient’s co-morbidity; therefore it would be possible to prescribe if physicians carefully determined the necessity. Extreme ageing cancer patients, primary lung cancer, metastatic disease at diagnosis, and unplanned hospitalizations were associated with shorter survival. Such vulnerable patients were among those who needed comprehensive geriatric assessment (CGA) to determine the most proper cancer care. Moreover, cancer patients with extreme age, metastatic disease at diagnosis and PP had strong tendency towards more unplanned hospitalizations. A physician should be more vigilant in taking care and prescribing such patients who are more likely to succumb to serious adverse events.

### Strengths

We conducted a prospective cohort study in a real-world situation and followed up the participants long enough to determine the adverse outcomes.

### Limitations

The investigators did not collect the data regarding socio-economic status and performance status. According to the latest evidences and systemic reviews, the socio-economic status was less clinical relevant to cancer treatment outcomes compared to other factors presented in this cohort study. Due to advances in cancer treatment and accessibility to the less toxic hormonal agents in some breast cancer patients and the epidermal growth factor receptor tyrosine kinase inhibitors in some lung cancer patients, patients with poorer performance status (ECOG PS 2) would gain benefits from active cancer treatment; therefore, the investigators recruited the participants solely based on an oncologist’s discretion. Moreover, this study determined PP in numerical basis only; however, duration of drug exposure would be a significant factor to elucidate the long-term association with adverse events.

### Supplementary Information

**Additional file 1.**

**Additional file 2. **

## Data Availability

The investigators sent the raw data and materials to the Editorials in Excel format. The raw data and statistical analytic models that support the findings of this study are available upon request from the corresponding authors.
