# Supplementary figures and images for: Polypharmacy and potentially-inappropriate medications are prevalent in the elderly cancer patients receiving systemic cancer therapy and they co-relate with adverse outcomes
Source: BMC Geriatr. 2023 Nov 27;23:775. doi: 10.1186/s12877-023-04471-3 (PMC10680314; doi:10.1186/s12877-023-04471-3)

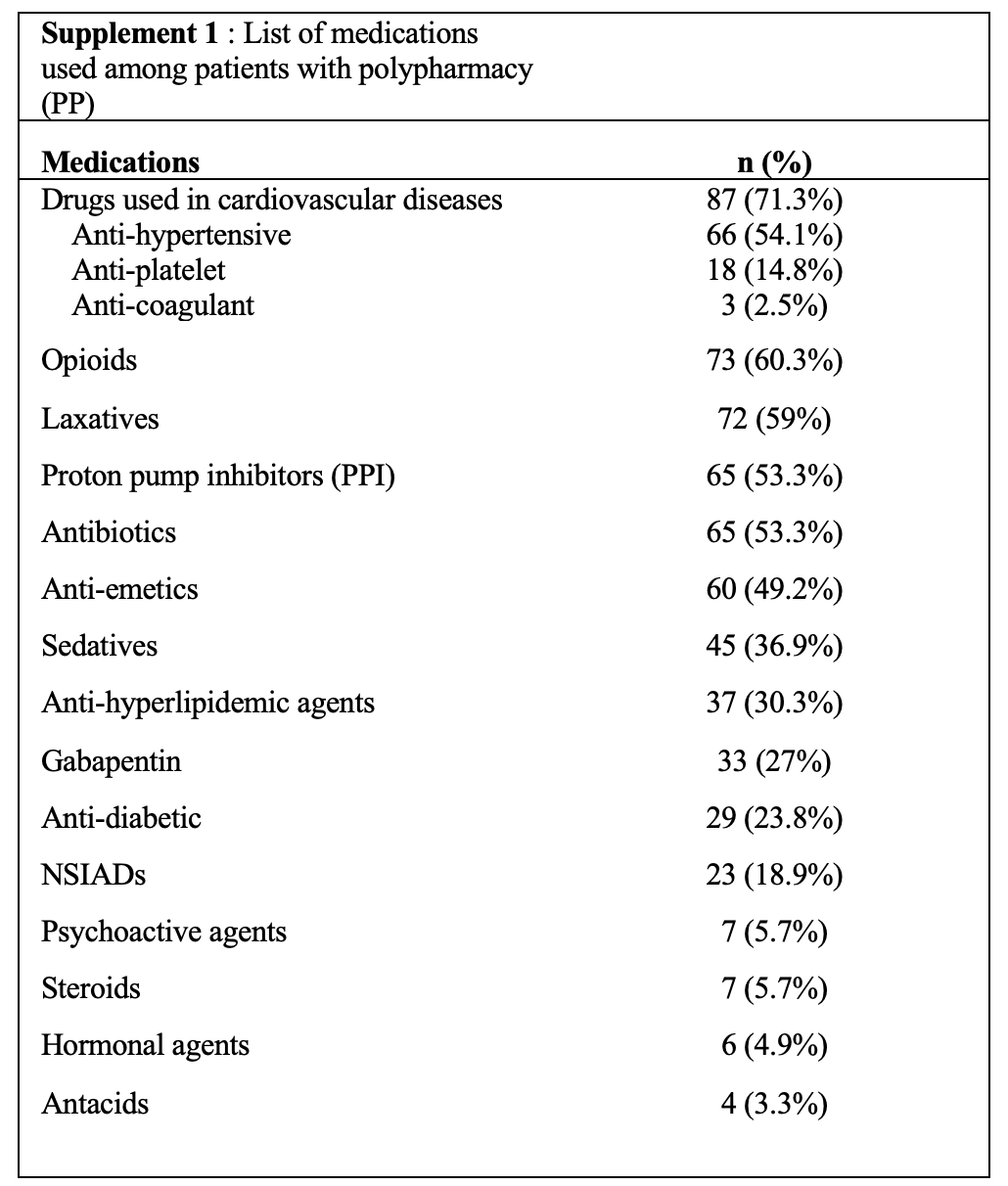

Supplement: Supplementary file 1 — Additional file 1. [file 12877_2023_4471_MOESM1_ESM.png]

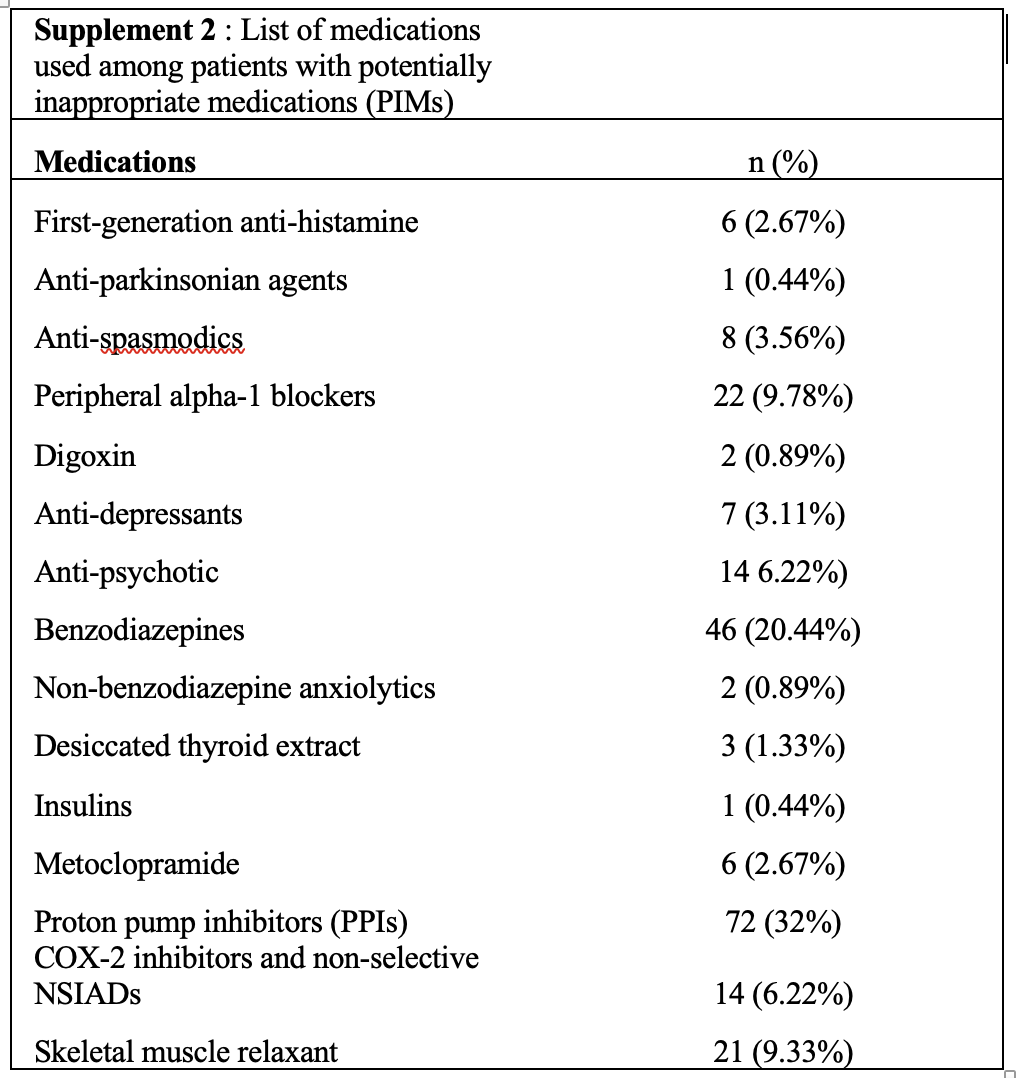

Supplement: Supplementary file 2 — Additional file 2. [file 12877_2023_4471_MOESM2_ESM.png]
